# Striatal correlates of Bayesian beliefs in self-efficacy in adolescents and their relation to mood and autonomy: a pilot study

**DOI:** 10.1093/texcom/tgad020

**Published:** 2023-11-02

**Authors:** Liana Romaniuk, Niamh MacSweeney, Kimberley Atkinson, Stella W Y Chan, Miruna C Barbu, Stephen M Lawrie, Heather C Whalley

**Affiliations:** Division of Psychiatry, University of Edinburgh, Kennedy Tower, Royal Edinburgh Hospital, Morningside Park, Edinburgh EH10 5H, United Kingdom; Division of Psychiatry, University of Edinburgh, Kennedy Tower, Royal Edinburgh Hospital, Morningside Park, Edinburgh EH10 5H, United Kingdom; Division of Psychiatry, University of Edinburgh, Kennedy Tower, Royal Edinburgh Hospital, Morningside Park, Edinburgh EH10 5H, United Kingdom; School of Psychology & Clinical Language Sciences, University of Reading, Earley Gate, Whiteknights, Reading RG6 6ES, United Kingdom; Division of Psychiatry, University of Edinburgh, Kennedy Tower, Royal Edinburgh Hospital, Morningside Park, Edinburgh EH10 5H, United Kingdom; Division of Psychiatry, University of Edinburgh, Kennedy Tower, Royal Edinburgh Hospital, Morningside Park, Edinburgh EH10 5H, United Kingdom; Division of Psychiatry, University of Edinburgh, Kennedy Tower, Royal Edinburgh Hospital, Morningside Park, Edinburgh EH10 5H, United Kingdom

**Keywords:** functional magnetic resonance imaging, reward learning, computational modeling, depression

## Abstract

Major depressive disorder often originates in adolescence and is associated with long-term functional impairment. Mechanistically characterizing this heterogeneous illness could provide important leads for optimizing treatment. Importantly, reward learning is known to be disrupted in depression. In this pilot fMRI study of 21 adolescents (16–20 years), we assessed how reward network disruption impacts specifically on Bayesian belief representations of self-efficacy (SE-B) and their associated uncertainty (SE-U), using a modified instrumental learning task probing activation induced by the opportunity to choose, and an optimal Hierarchical Gaussian Filter computational model. SE-U engaged caudate, nucleus accumbens (NAcc), precuneus, posterior parietal and dorsolateral prefrontal cortex (P_FWE_ < 0.005). Sparse partial least squares analysis identified SE-U striatal activation as associating with one’s sense of perceived choice and depressive symptoms, particularly anhedonia and negative feelings about oneself. As Bayesian uncertainty modulates belief flexibility and their capacity to steer future actions, this suggests that these striatal signals may be informative developmentally, longitudinally and in assessing response to treatment.

## Introduction

Major depressive disorder (MDD) is a common disorder with an estimated lifetime risk of 15%–18% (Bromet et al. 2011; Malhi and Mann 2018), which can impose significant suffering and functional impact across the lifespan. It often has its roots in adolescence, with peak incidence between ages 15–20, and 25% of individuals having been diagnosed by age 19, (Kessler et al. 2005; McGrath et al. 2023). Although treatments are available, their mechanisms of effect remain only partly understood, and full remission after a first episode is seen in only 50% of patients (Rush 2007). It is therefore key that we develop a more mechanistic understanding of depression that highlights potential ways forward regarding treatment.

One brain system of particular interest concerns reward processing (Admon and Pizzagalli 2015), which demonstrates prominent subcortical responses in adolescents versus adults (Silverman et al. 2015), and gradually shifts to more frontal regions during maturation (Schreuders et al. 2018; Willinger et al. 2021). The reward system also shows evidence of dysfunction in adolescent MDD (O’Callaghan and Stringaris 2019). Treatment response can be significantly predicted by levels of anhedonia and motivation (Uher et al. 2008; McMakin et al. 2012), which encompasses the anticipation, effort expenditure and consummatory experience of reward (Rizvi et al. 2016; Keren et al. 2018).

However, it is important to consider how such dysfunction can exert its effects most problematically. Here, we propose that reward network dysfunction within the context of self-efficacy is of particular relevance to MDD. Self-efficacy is the belief in your own ability to plan and execute goal-directed behavior^13^, and has been found to mediate the relationship between risk factors such as adverse childhood experiences and current quality of life in young adults (Cohrdes and Mauz 2020). It is associated with increased beneficial activity and cognitive self-care strategies in the context of recovery from depression (McCusker et al. 2016). The mental representations that we construct regarding ourselves and our capabilities must be flexibly responsive to our experiences (Price and Duman 2020; Koban et al. 2021). Adolescence is a period of dramatic neurobiological change (Dahl 2004; Blakemore 2012), particularly concerning prefrontal synaptic overproduction and pruning (Petanjek et al. 2011), which coincides with the greatest levels of gene expression regulating neuronal development (Harris et al. 2009). Age-related shifts from forward to backward projections within the reward system are argued to energize the exploratory behavior that is so important in allowing adolescents to develop self-efficacy and autonomy (Luciana et al. 2012). Self-referential networks (Pfeifer and Peake 2011; Ernst et al. 2019) are particularly dynamic, reflecting that this is a time when we are actively developing beliefs and models about oneself and capabilities (Crone and Dahl 2012; Harter 2012), with wide-ranging future implications. If the means to evaluate and incorporate the consequences of our actions via the reward system becomes compromised, then this could give rise to an increasingly entrenched and biased model of the self (Hoven et al. 2019; Kopala-Sibley and Zuroff 2020).

A negatively-skewed inner model of self-efficacy during adolescence can lead to a vicious circle of lost opportunities and deepening bias. These skewed beliefs could impair motivation and social engagement, causing functional debilitation by depriving people of the sense that their opportunities are actually available to them (Milanovic et al. 2018). However, this also makes adolescence a time of potential opportunity, as therapeutic approaches that directly challenge a skewed internal working model may confer longer-term benefits.

Neuroimaging, particularly when used in concert with computational approaches, has the power to test mechanistic hypotheses of such phenomena. Functional MRI (fMRI) has been used to explore several aspects of the self, including self-esteem (Chavez and Heatherton 2015; Will et al. 2017; van der Aar et al. 2019), sense of agency (Haggard 2017; Beyer et al. 2018), and self-appraisal/valuation (Denny et al. 2012; Brühl et al. 2014; van der Cruijsen et al. 2019; Peters et al. 2021; van Buuren et al. 2022), consistently implicating a self-referential brain network comprising the precuneus, medial and dorsolateral prefrontal cortex, anterior cingulate and the striatum. To date, there have been few neuroimaging studies looking specifically for brain correlates of self-efficacy. A recent example applied Rescorla-Wagner models to account for fMRI responses akin to self-esteem, finding that self-esteem belief update signals correlated with activity in ventromedial prefrontal cortex (vmPFC) (Will et al. 2017). Rouault and Fleming (Rouault and Fleming 2020) have defined the concept of global self-performance estimates, which integrates more momentary and task-specific local estimates, and provides an account for fMRI activation within ventromedial prefrontal cortex and precuneus. Notably, this global measure—most akin to general self-efficacy—was particularly aligned with activity within ventral striatum.

Extending on the capabilities of Rescorla-Wagner models, Hierarchical Gaussian Filters (HGF) offer a means to formally represent the formation and updating of Bayesian beliefs over a series of increasingly abstract hierarchies (Mathys et al. 2014), using the mismatch between expectations and experience (prediction error) to update these evolving beliefs, weighted according to their associated precision, otherwise known as certainty or confidence. Prior beliefs with high precision require greater evidence to be updated toward more accurate posterior beliefs. High uncertainty signals could represent a more malleable belief system, whereas low uncertainty implies a system that is robust to change. Both states have the potential to be adaptive or maladaptive, in part dependent on whether the beliefs are represented within limbic versus association/cognitive networks (Heinz et al. 2018). HGF have been successfully applied to fMRI data in the context of social beliefs (Henco et al. 2020), but they have yet to be applied in considering self-efficacy.

Based on the above, we carried out a pilot fMRI study to examine brain correlates of self-efficacy beliefs (SE-B) and their associated uncertainty (SE-U) within a sample of young people experiencing depressive symptoms. Using the Modified Inherent Value of Choice task (MIVCT), we examined neural activation related to anticipation of being able to make your own choice, compared to passively obeying what the computer instructs you to do. HGF modeling was applied to implicitly estimate each participant’s evolving self- and other-efficacy beliefs, based on their behavior and patterns of received rewards. Our hypotheses tested whether one’s estimates of self-efficacy (a) covary with activation within the brain’s reward and salience networks, and (b) that this in turn demonstrates relationships with current depressive symptoms and measures related to one’s sense of autonomy (Deci and Ryan 1985), applying partial least squares analysis to account for multicollinearity.

## Materials and methods

### Participants

This study was part of a pilot that sought to develop and trial three novel fMRI paradigms within a cohort of late-adolescents/young adults, with a focus on feasibility, tolerability, and establishing activation signals for future power calculations. The other two tasks will be analyzed separately. Thirty young people aged 16–20 years were recruited from the community via posters and a local social media campaign, facilitated by third sector organizations, schools (with local educational authority approval), colleges and universities in the region. Inclusion criteria were self-reported history of depressive symptoms or an episode; to be able to provide informed consent; normal/corrected-to-normal vision and hearing; self-reported fluency in English; and no contraindications for MRI. Exclusion criteria were a clinical diagnosis of a pervasive developmental disorder; neurological or genetic disorder; or intellectual disability. The study had ethical approval (REC number 19-HV-061) and participants provided written informed consent. Participants’ travel costs were reimbursed, and they received a picture of their brain as a token of gratitude for taking part.

### Clinical and psychological measures

Depressive symptom severity was assessed using the Patient Health Questionnaire (PHQ-9 (Kroenke et al. 2001)), a validated self-report measure based on DSM-IV MDD criteria. It examines the nine key symptom groups of anhedonia, dysthymia, sleep disturbance, anergia, appetite disturbance, poor self-esteem, poor concentration, psychomotor disturbance and self-harm/suicidal thoughts, each rating current severity from 0 to 3. Total scores > = 10 have sensitivity 88% and specificity 88% for MDD (Kroenke et al. 2001). We also incorporated measures of relevance to one’s sense of self-efficacy and autonomy, specifically (a) the General Causality Orientations Scale (Deci and Ryan 1985) adapted for clinical populations (GCOS-CP (Cooper et al. 2015)) which measures the degree to which the source of one’s motivation and behavioral initiation comes from within (Autonomy subscale), from without (Control orientation) or is indeterminate (Impersonal orientation), as defined by Self-Determination Theory (Ryan and Deci 2017); (b) the Perceived Choice and Awareness of Self Scale (PCASS (Sheldon and Deci 1996)), which measures how much choice you have over your own behavior (Perceived choice, PC) and awareness of your inner states/sense of self (Awareness of self, AOS); and finally the Brief Resilience Scale (BRS (Smith et al. 2008)), which measures the ability to recover from adversity.

### Imaging protocol

MRI scanning took place at the Clinical Research Imaging Centre (CRIC), University of Edinburgh, using a 3T Siemens Magnetom Skyra scanner and 32-channel head coil. A T1-weighted MPRAGE structural image was acquired having 192 contiguous 1.0 mm slices (matrix = 256 × 256; FoV = 256 mm; TR = 2.5 s, TE = 4.37 ms, flip angle = 7°). BOLD signals were acquired using an axial gradient echo planar imaging (EPI) sequence with TR = 1.4 s, TE = 30 ms, flip angle = 68° and 2× GRAPPA acceleration. FoV = 210 mm, spatial resolution 3 mm isotropic.

### Modified inherent value of choice task (MIVCT)

**Fig. 1:**
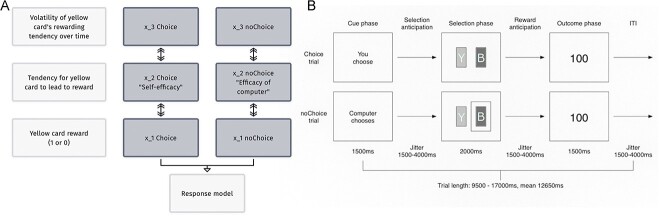
A) Overall schema of the hierarchical Gaussian filter, in this case displaying three layers, with parallel models for the choice and noChoice conditions. The second level represents the tendency for the yellow card to be rewarding in the choice (i.e. self-efficacy) and noChoice (other-efficacy) contexts. For two-layer models, the highest layer representing volatility is omitted. B) The modified inherent value of choice task (MIVCT).

The MIVCT is an instrumental reward learning task that probes the value participants place on being able to make decisions themselves (Fig. 1). It was adapted (Leotti and Delgado 2011) and described in previous studies (Romaniuk et al. 2019; Rupprechter et al. 2020). In brief, participants learn by trial and error which of two-color stimuli (yellow and blue) lead to a reward (80:20% reinforcement). For half of the trials, they get to choose (Choice condition), and for the other half, they must do as the computer directs them (noChoice condition). Stimuli reward contingencies remained consistent across Choice and noChoice. During the scan, the task was implemented using Presentation (https://www.neurobs.com). Each trial is divided into three phases: the Cue phase informs participants whether this will be a Choice or noChoice trial; the Selection phase is when the yellow or blue stimulus is selected; and the Outcome phase when the participant finds out their reward. The three phases are jittered to permit their disambiguation during event-related fMRI analysis. There were 27 trials for each condition. Trial order and the side of the screen on which stimuli were presented were randomized to prevent action planning. noChoice trial decisions echoed the participant’s own choices, with a three-trial lag, to ensure rewards were matched between conditions. Task duration was 11 m 16 s, 483 volumes.

### Hierarchical Gaussian filter modeling

The HGF framework was used to implement five related models, with Bayesian model selection applied to determine that which provided the best account for the data. Each model permits inferences to be made regarding the participants’ emerging beliefs about their own tendency to be correct, versus that of the computer, based on their behavioral data. Similar frameworks have been successfully applied within several neuroimaging studies to date (Iglesias et al. 2013; Deserno et al. 2019; Henco et al. 2020). It was implemented using the TAPAS HGF toolbox version 6 (Frässle et al. 2021). Each model comprised a binary perceptual model, and a response model. The binary perceptual model estimates each participant’s beliefs about the hidden states of their sensory inputs over time. The response model describes how the participant’s actions are informed by these beliefs.

The perceptual model was composed of two parallel models, with separate hierarchies for Choice and noChoice trials (Fig. 1). Both are inversions of generative models of the participant’s sensory experiences, which in this context are card choice and subsequent reward. The generative model attempts to infer the hidden states of the environment $x$, by updating the posterior probability distributions of a hierarchical series of beliefs μ, which are updated after each trial. Two variants of perceptual model were tested, comprising of two or three hierarchical levels. The first and lowest level ${x}_1$ is a binary representation of whether or not the yellow card was associated with a reward. The second level ${x}_2$ represents the tendency for the yellow card to be rewarding. For three-level models, the third and highest level ${x}_3$ represents the volatility of this tendency over time. Given the parallel architecture, the beliefs represented at the intermediate level ${x}_{2\ Choice}$ were analogous to the tendency for yellow to be rewarding in the context of the self making the choice, i.e. self-efficacy or μ_self_, as opposed to the computer making the choice ${x}_{2\ noChoice}$, i.e. other-efficacy or μ_other_. The trial-by-trial variance associated with each belief trajectory were also estimated: ${\hat{\sigma}}_{2\ Choice}^k$ i.e. the uncertainty around the self-efficacy belief or σ_self_, and ${\hat{\sigma}}_{2\ noChoice}^k$, the corresponding uncertainty around other-efficacy or σ_other_.

${x}_2$ and ${x}_3$ develop as Gaussian random walk processes, with step-sizes defined by their evolution rates ${\omega}_{2\ Choice}$, ${\omega}_{2\ noChoice}$, (which describe how quickly the contingencies between card and outcomes evolve for each participant, irrespective of phasic changes), and for three-level models ${\omega}_{3\ Choice}$ and ${\omega}_{3\ noChoice}$, which are the equivalent for the volatilities. ${x}_2$ is sigmoid-transformed to produce a probability of ${x}_1$.The model is then inverted according to the card chosen and reward received for each trial $k$, in order to infer each participant’s evolving hierarchical beliefs over the course of the task. Beliefs at each hierarchical level $i$ are updated according to the prediction error from the level below ${\delta}_{i-1}^k$, which is weighted by the ratio of the precisions $\pi$ of the beliefs at $i$ and $i-1$. A belief’s precision is the inverse of its variance ${\sigma}_i^k$, i.e. ${\pi}_i^k=1/{\sigma}_i^k$, defined as:


$$ {\hat{\pi}}_i^k=\frac{1}{{\hat{\mu}}_i^k\left(1-{\hat{\mu}}_i^k\right)} $$

Beliefs are updated according to:


$$ \varDelta{\mu}_i^k=\frac{\pi_{i-1}^k}{\pi_i^k}{\delta}_{i-1}^k $$

The response models also had parallel Choice and noChoice architectures. Two variants were tested: a softmax model, which did not depend on belief certainty; and a unit square sigmoid model, the slope of which was defined by how certain the participant was estimated to be in their own (or the computer’s) tendency to be correct.

For the softmax model, The probability of choosing yellow was


$$ {prob}_{yellow}^k=\frac{1}{\Big(1+\mathit{\exp}\left(-\beta{\hat{\mu}}_1^k\right)} $$

Where free parameter $\beta$ is the participant’s decision noise.

For the unit square sigmoid model, the probability of choosing yellow was


$$ {{prob}_{yellow}^{k}} = \frac{{{\hat{\mu}}_{1}^{k}}{}^{{\zeta}^{k}}}{\left({{\hat{\mu}}_{1}^{k}}{}^{{\zeta}^{k}} + (1- {{\hat{\mu}}_1^k}){}^{\zeta^k}\right)} $$

Where ζ^k^ defines the shape of the sigmoid for each trial k, which in this case was set to $-\log \left({\hat{\sigma}}_{2\ Choice}^k\right)$ or $-\log \left({\hat{\sigma}}_{2\ noChoice}^k\right)$: the negative log-transformed variance regarding the tendency for the participant or computer to be correct, respectively.

A simple Rescorla-Wagner model with fixed learning rate and softmax response model was also tested as a control, to establish whether the HGF framework in itself provided an improved account for the data.

Trial-by-trial estimate trajectories of μ_self_, μ_other_, σ_self_, and σ_other_ were derived by fitting the model to each participant’s card selections and associated rewards. Free parameters during model fitting were the evolution rates ${\omega}_{2\ Choice}$, ${\omega}_{2\ noChoice}$; 3-level models also incorporated ${\omega}_{3\ Choice}$ and ${\omega}_{3\ noChoice}$; and models using the softmax response model included decision noise parameters ${\beta}_{Choice}$ and ${\beta}_{noChoice}.$ Random-effects Bayesian model selection was used to select the optimal model of the five possible architectures, according to their negative variational free energy (approximating log model evidence). That with the highest protected exceedance probability was deemed optimal (Stephan et al. 2009). The winning model was then tested to see whether the percentage of sufficiently-explained trials was better than chance, i.e. that the negative log likelihood per trial exceeded 0.55 (Reiter et al. 2017).

### Functional imaging pre-processing and analysis

fMRI data were preprocessed and analyzed using SPM12 (http://www.fil.ion.ucl.ac.uk/spm/software/spm12/), within MathWorks MATLAB R2022a (http://www.mathworks.com). fMRI volumes were reconstructed into NIfTI format, and realigned to the mean volume. The structural image was segmented and warped to MNI space. The mean fMRI and structural volumes were co-registered, and the normalization parameters applied to the whole fMRI dataset which was then smoothed using a 6 mm FWHM gaussian kernel, and resampled at 2 mm isotropic resolution. The data was high pass filtered with 128 s cutoff, and serial correlations modeled using a first-order autoregressive model.

For each participant, and following optimal HGF model fitting to their behavioral data, the estimated efficacy belief trajectories ${\mathrm{\mu}}_{\mathrm{Self}}$ and ${\mathrm{\mu}}_{\mathrm{Other}}$ were used to parametrically modulate the moment when participants learned whether or not they would be doing the choosing, during Choice and noChoice trials respectively within a first-level GLM. As μ and σ estimates were correlated, the uncertainty trajectories σ_self_, and σ_other_ were considered in a separate first-level GLM, modulating the same moment of Choice and noChoice trials. Motion parameters were included as nuisance regressors. Contrasts representing differences in the beliefs of self- versus other-efficacy (SE-B: μ_self_ > μ_other_), and the uncertainty surrounding those beliefs (SE-U: σ_self_ > σ_other_) were evaluated within second-level F tests. Activation was deemed significant if it achieved cluster-level familywise-corrected significance of p < 0.05 for the whole brain volume. This task-defined activation was extracted as first eigenvariates from these clusters for subsequent analyses.

### Associations between brain activation, psychometrics and symptoms

Sparse partial least squares regression (sPLS) was used to identify the relationships brain correlates of SE-B and SE-U related to expressed psychology and psychopathology, while accounting for multicollinearity. Using the MixOmics toolbox in R (Cao et al. 2016; Rohart et al. 2017), brain activations extracted from significant clusters were regressed onto each PHQ9 depressive symptom, GCOS-CP Autonomy, Control and Impersonal subscales; the PCASS PC and AOS subscales and the BRS resilience score. A two-step optimization process was used, whereby first the optimal number of variates to be included, and then the most stably-selected variates were identified, using 4-fold cross validation over 1,000 repeats. Statistical tests were performed within confirmatory regressions on those subsets implicated by sPLS, using the MASS toolbox within R (Venebles and Ripley 2002). Brain regions implicated by sPLS were also regressed on each symptom of the PHQ-9, using backwards stepwise regression, with an optimal model selected according to Akaike information criterion (AIC).

## Results

### Demographics and clinical measures

Of the original 30 participants, four were excluded as they didn’t respond during noChoice trials; and five were excluded due to excessive motion (any single volume-to-volume displacement > 1 mm), leaving 21 participants for analysis. Participant characteristics are described in Table 1. The excluded participants did not differ from the included group in terms of age, or PHQ-9, GCOS-CP, PCASS or BRS scores (p > 0.209). The 21 included participants had PHQ-9 scores ranging between 3 and 24, with mean 11, signifying depression of moderate severity. Two were currently taking antidepressants (fluoxetine and sertraline). PHQ-9 depression scores significantly correlated with GCOS-CP Autonomy (r = −0.510, p = 0.018), Impersonal (r = 0.449, p = 0.041), PCASS Awareness of Self (r = −0.526, p = 0.014) and Perceived choice (r = −0.630, p = 0.002). The estimated computational model parameters can be found in the supplementary materials.

**Table 1:** The demographic, computational and clinical characteristics of 21 included participants. Computational model estimated parameters are reported for the optimal model, specifically the 3-layer HGF with uncertainty-dependent response model.

	Measure	Mean (SD)
	Age	18.9 (0.825)
	Sex F:M	14: 7
	PHQ-9 Depression Severity: None Mild Moderate Moderately-severe Severe	11.0 (6.05) N: 1 9 5 3 3
	Taking antidepressant Yes: No	2: 19
	GCOS-CP Autonomy	43.7 (5.33)
	GCOS-CP Control	25.1 (7.08)
	GCOS-CP Impersonal	30.5 (6.76)
	PCASS Awareness of Self	14.4 (4.93)
	PCASS Perceived Choice	15.8 (4.30)
	BRS resilience	3.17 (0.32)
Optimal perceptual model evolution rates	${\omega}_{2\ Choice}$	−6.10 (2.21)
	${\omega}_{2\ noChoice}$	−6.33 (0.978)
	${\omega}_{3\ Choice}$	−6.00 (0.019)
	${\omega}_{3\ noChoice}$	−6.00 (0.001)

### Computational model selection

The optimal model comprised the 3-layer HGF perceptual model, and the response model whose unit square sigmoid was modulated by belief uncertainty (${\hat{\sigma}}_{2\ Choice}^k$ and ${\hat{\sigma}}_{2\ noChoice}^k$), with protected exceedance probability 0.848 (Table 2), clearly outperforming the alternatives. For each participant, this winning model performed better than chance at accounting for behavior, with negative log likelihood per trial exceeding 0.55 in all cases (sample median 0.662, IQR 0.045). Model estimated parameters ${\omega}_{2\ Choice}$, ${\omega}_{2\ noChoice}$, ${\omega}_{3\ Choice}$, and ${\omega}_{3\ noChoice}$, as well as their respective Choice vs noChoice differences, did not significantly correlate with any psychometric score (p > 0.182, Supplementary Table S1) or age (p > 0.302).

**Table 2:** Bayesian model selection between the five proposed computational models.

Perceptual model	2-layer HGF		3-layer HGF		Rescorla-Wagner
Response model	Softmax	Uncertainty-dependent	Softmax	Uncertainty-dependent	
Posterior model probability	0.079	0.317	0.063	0.500	0.042
Exceedance probability	< 0.001	0.145	< 0.001	0.854	0.002
Protected exceedance probability	0.002	0.145	< 0.002	0.848	0.001

### SE-B belief in self- versus other-efficacy

The SE-B F test of μ_self_ > μ_other_ demonstrated no significant activation at the whole-brain level.

### SE-U uncertainty around belief in self- versus other-efficacy

**Fig. 2:**
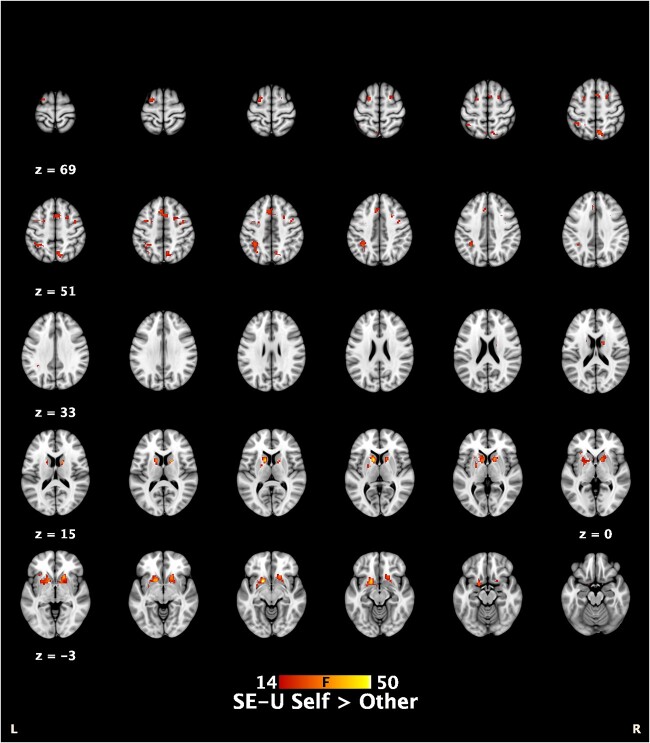
Brain activation covarying with SE-U, where in cases self > other. Areas displayed demonstrate cluster-wise whole-brain significance of p < 0.05, FWE-corrected.

The SE-U F test examining the uncertainty around the beliefs of Self-efficacy > Other-efficacy (σ_self_ > σ_other_) showed significant whole-brain activation in bilateral anterior striatum including NAcc; bilateral dorsolateral prefrontal cortex (dlPFC); midline cortex including supplementary motor (SMA) and precuneus; and posterior parietal cortex (see Table 3, Fig. 2). In all cases, the direction of effect was σ_self_ > σ_other._

**Table 3:** Whole-brain significant activation for the SE-U F contrast of uncertainty around belief in self-efficacy > other-efficacy σ_self_ > σ_other_. MFG: middle frontal gyrus; SFG: superior frontal gyrus; SMA: supplementary motor area; SMG: supramarginal gyrus; SPL: superior parietal lobule.

Region	Peak MNI	Peak Z	k_E_	Cluster P_FWE_
L Putamen/nucleus accumbens	−18 8 −10	4.82	292	< 0.001
L Caudate	−12 8 5	4.80		
L Putamen/anterior insula	−27 14 −1	4.03		
R Caudate	18 8 11	4.52	192	< 0.001
R Putamen/nucleus accumbens	18 17 −4	4.52		
R dorsal MFG/SFG	27 11 41	4.08	62	0.005
L & R SMA	−6 20 47	3.98	108	< 0.001
L SPL/SMG/angular gyrus	−33 −49 41	3.97	130	< 0.001
L & R precuneus	9 −67 53	3.92	82	0.001
L dorsal MFG/SFG	−36 2 47	3.80	104	< 0.001

To provide further evidence for the specificity of the winning computational model (3-layer HGF with uncertainty-dependent response model) in accounting for brain activation, the Rescorla-Wagner’s estimates of the value $\hat{V}$ of the Choice and noChoice trials was also estimated and used to parametrically modulate the Cue phase of each trial. The contrast of ${\hat{V}}_{Choice}>{\hat{V}}_{noChoice}$ showed some activation in midline and striatal areas, but to a much less degree than σ_self_ > σ_other_ (Supplementary Materials).

### Multivariate regression of psychometric measures and symptoms

Having extracted the first eigenvariate from each of the significant SE-U σ_self_ > σ_other_ cluster regions, sPLS regression was used to identify relationships with the psychometrics of interest, for both Self (Choice) and Other (noChoice) conditions.

**Fig. 3:**
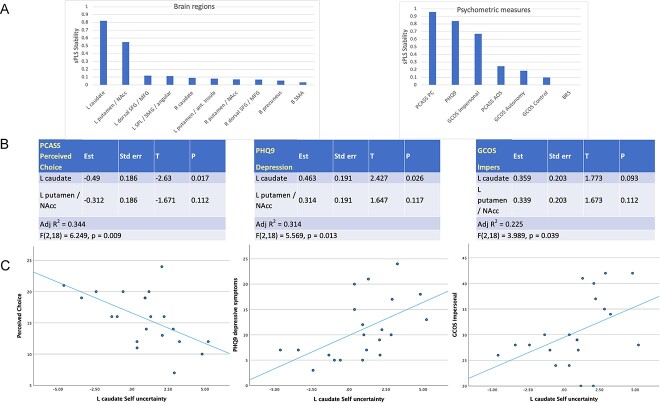
The relationships between self-efficacy uncertainty and psychometric measures. A) The stability of how frequently each variate was selected for the optimal sPLS regression over cross-validation. B) Confirmatory regressions of self-efficacy uncertainty-associated brain activation on sPLS-implicated psychometric measures. C) Scatter plots demonstrating the relationships between L caudate self-efficacy uncertainty activation and implicated psychometric measures.

For Self-efficacy uncertainty, an optimal correlation between selected variates and the full model was achieved with two brain regions, and three psychometric measures, accounting for 0.401 of the variance across psychometrics. The two most stably-selected regions were L caudate and L putamen/NAcc; the three most-stable psychometrics were PCASS Perceived Choice, PHQ-9 depression and GCOS Impersonal (Fig. 3A). Confirmatory stepwise regression of brain activation on each psychometric measure highlighted the prominent relationships between L caudate Self-efficacy uncertainty and both PHQ-9 depression (p < 0.026), and PCASS Perceived choice scores (p < 0.017, Fig. 3B). A stepwise regression of PHQ-9 depressive symptoms on L caudate SE-U activation produced an optimal model that comprised anhedonia, negative feelings about the self, sleep disturbance, poor concentration and feeling tired (Table 4).

**Table 4:** Stepwise regression of PHQ-9 depressive symptoms on L caudate SE-U activation.

PHQ9 Item	Est	Std err	T	P
Anhedonia	0.723	0.171	4.23	< 0.001
Feel bad about self/guilt	0.522	0.151	3.45	0.004
Sleep disturbance	0.344	0.180	1.91	0.075
Poor concentration	−0.324	0.172	−1.89	0.078
Feeling tired	−0.312	0.180	−1.56	0.140

Adj R^2^ = 0.605. F(5,15) = 7.13, p = 0.001.

For Other-efficacy uncertainty, the optimal sPLS comprised two brain regions (B SMA and R caudate), and five psychometrics (PCASS Awareness of Self, PCASS Perceived Control, BRS, PHQ-9 and GCOS Control), accounting for 0.345 of psychometric variance, however none of the confirmatory regressions achieved significance (p > 0.069).

## Discussion

In this pilot study exploring how computational models of self-efficacy can relate to brain function within a group of adolescents with self-reported low mood, we find evidence that activation within several brain regions, particularly striatal reward areas, can be accounted for by a three-layer HGF computational model characterizing beliefs around self-efficacy and their associated uncertainty. In particular, activation representing the uncertainty surrounding beliefs in self-efficacy within L caudate covaried with measures of perceived choice, and having an impersonal causality orientation, providing face validity. This activation also demonstrated potential clinical utility, through its associate with the severity of current depressive symptoms, particularly anhedonia and negative feelings about the self.

### Belief uncertainty, reward networks and depression

Dysfunction within reward networks is a well-replicated finding within people with depression (Keren et al. 2018; Fox and Lobo 2019). However, corticostriatal circuits (Peters et al. 2016) and their dopaminergic innervation are implicated in multiple mental disorders, including attention-deficit hyperactivity disorder (Plichta and Scheres 2014), eating disorders (Val-Laillet et al. 2015), psychosis (McCutcheon et al. 2019), obsessive-compulsive disorder (Robbins et al. 2019) as well as affective disorders (Gong et al. 2020). This pervasive presence of striatal dysfunction across diagnoses may be due to it being well-placed to mediate the updating of beliefs across broader cortical networks (Broyd et al. 2017; Nour et al. 2018; Gershman and Uchida 2019) via the partially-overlapping hierarchically spiraling loops that reconnect it to the cortex via the thalamus (Haber 2016). Understanding the specific nature and contribution each pattern of striatal dysfunction makes to an individual’s current state could therefore be highly informative, particularly in view of the pharmacotherapeutic potential for intervention at this site. Here, we focused on how such dysfunction might be relevant in the context of depression, by modeling one’s beliefs about self-efficacy, and find that L caudate self-efficacy uncertainty relates to negative beliefs about the self, as well as one’s sense of how much choice they have over their own behavior.

Within a Bayesian framework, belief uncertainty will impact on not only how readily beliefs can be updated by new evidence, but also how influential they are in guiding behavior, according to active inference (Knolle et al. 2023). Conceptually, self-efficacy represents a bridge between self-referential processes and behavior, providing a means by which self-related beliefs can impinge on a person’s inclination to act. If one is more certain in their own self-efficacy, then if follows that they will be more likely to take action. The specific relationship with anhedonia could be a consequence of a more confident belief underpinning a deeper sense of personal involvement and connection to the act (Yeshurun et al. 2021) It is noteworthy that anhedonia is higher in those with impaired connectivity between striatum and several of the cortical regions we report to correlate with self-efficacy uncertainty (Gabbay et al. 2013). A broader network analysis of depressive symptoms has found that all five of the symptoms associated with L caudate self-efficacy uncertainty feature prominently in the most-central symptoms out of a possible 28 (Fried et al. 2016). Lack of energy and anhedonia also make a substantial contribution to the functional impairment seen in depression (Fried and Nesse 2014). This signal could therefore be broadly informative across the range of phenotypic variation commonly observed under the umbrella of depression.

Anatomically, this caudate region appears to play a particular role in positively- and negatively valanced beliefs about the self: is connected to anterior medial prefrontal and inferior frontal/insula regions (Fan et al. 2016), which together are often activated by aversive stimuli (Kober et al. 2008) as part of a proposed negative affect circuit (Williams 2016). Corticostriatal tract integrity between these regions can also predict self-esteem (Chavez et al. 2022). These findings therefore provide preliminary support for L caudate being a site where belief uncertainty signals of specific relevance to depression are meaningfully detectable. Although requiring replication in a larger sample, such a signal could guide optimal pharmacotherapy, by identifying those with markedly affected belief-updating networks, and tracking improvements in response to treatment. It has been observed that those with abnormally low striatal reward responses go on to experience the most benefit from the serotonin (and weak dopamine)-reuptake inhibitor sertraline (Greenberg et al. 2020) and noradrenaline-dopamine reuptake inhibitor bupropion (Nguyen et al. 2019). The link between striatal responses and belief uncertainty may be an additionally informative step, and lays the foundations to maximize the efficacy of psychological therapies intended to improve cognitive flexibility and the adoption of more adaptive, less pathologically-negative beliefs (Shany et al. 2022).

### Self-efficacy, adolescence and mental illness

In this study we have used each participant’s task behavioral data to implicitly estimate their underlying beliefs regarding self-efficacy, rather than asking them to explicitly rate themselves. This has the potential advantage of accessing the parameter in itself, rather than the “meta-parameter” of one’s publicly-acknowledged assessment of their own self-efficacy. Self-efficacy is of key relevance to all mental disorders, especially if considered within the broader concept of autonomy. Autonomy, which encompasses the dimensions of competence (the ability to form goals and act on them) and authenticity (how one personally endorses their motivations to act) is undermined in all mental illnesses, albeit in different ways (Bergamin et al. 2022). Indeed, this interference with or loss of autonomy could be argued to be what defines a mental illness as such, regardless of its associated clusters of signs and symptoms. To have a better understanding of the neurobiology underpinning autonomy could therefore have a broader clinical relevance. A developing sense of autonomy and self-efficacy are especially important hallmarks of adolescence, so this period of life could afford us the opportunity to observe how the neurobiology of self-efficacy evolves through adolescence longitudinally. It is known that striatal responses to positive > negative self-evaluations show an inverted-U curve with age, being lowest though adolescence (van der Cruijsen et al. 2018). Longitudinally, young adults with low self-directedness have higher depressive scores three years later (Takanobu et al. 2021). This is evidence for the potential impact of this phase of life on the developing sense of self, and it is hoped that this study can lead into a larger-scale longitudinal cohort study of young people on the cusp of adolescence, allowing us to track how these fMRI- and computationally-derived signals of self-efficacy develop over time, particularly in relation to symptom and functional trajectories.

### Self-referential processing and the default mode network

SE-U engaged a range of cortical areas, including dorsal superior frontal gyri, medial prefrontal cortex, angular gyri and precuneus. These are all considered part of the default mode network (DMN (Raichle 2015)), which contributes to internally-oriented processes including self-referential processing (Northoff et al. 2006; Qin and Northoff 2011), and has been reliably found to demonstrate reduced connectivity in depression (Tozzi et al. 2021). The precuneus is particularly relevant with regard to self-appraisal: its activity is positively correlated with academic self-regard in adolescents (van der Aar et al. 2019); and estimates of general self-efficacy correlate with its volume (Sugiura et al. 2016). The connectivity it demonstrates with dorsal PFC and angular gyrus is increased in those with depression (Cheng et al. 2018). However, using a multivariate approach, these cortical regions did not demonstrate a prominent relationship with the psychometric measures of interest, above and beyond that provided by L striatum. This isn’t to say that they don’t play a part, but within this pilot sample, most variance was accounted for by striatal activation.

### Strengths and limitations

This study has attempted to apply theoretically-grounded computational models to functional data in a principled way, using Bayesian model selection to identify the optimal model given the evidence. Regions demonstrating significant covariation with model-derived estimates were then related to psychometric and symptoms measures within a multivariate framework that accommodated multicollinearity.

The implicit nature of how self-efficacy beliefs and uncertainty were estimated in this work would benefit from additional validation within longitudinal studies. This was a pilot study involving a small sample of self-selected young people, and although their mean PHQ-9 score suggested clinically-significant psychopathology, this was not a formal clinical sample. They were also predominantly female, and it could be informative to specifically address sex differences in the context of self-efficacy and depression.

## Conclusions

Here we have shown that computational estimates of belief uncertainty regarding self-efficacy implicitly derived from behavioral responses during a reward task provide an account for patterns of brain activation in areas key to the reward system. This in turn correlates both with depressive symptoms and perceived choice in a group of adolescents. These signals could be informative in future longitudinal studies of development, as well as in assessing response to treatment for depression.

## Supplementary Material

issf_reward_paper_supplementary_materials_tgad020

## Data Availability

Anony mised data can be made available via the corresponding author upon request.
