# Supplementary material for: Striatal correlates of Bayesian beliefs in self-efficacy in adolescents and their relation to mood and autonomy: a pilot study
Source: Cereb Cortex Commun. 2023 Nov 2;4(4):tgad020. doi: 10.1093/texcom/tgad020 (PMC10712445; doi:10.1093/texcom/tgad020)
Supplement: issf_reward_paper_supplementary_materials_tgad020 [file issf_reward_paper_supplementary_materials_tgad020.docx]

Supplementary materials

Striatal correlates of Bayesian beliefs in self-efficacy in adolescents and their relation to mood and autonomy: A pilot study

# Results

## Correlations between clinical, psychological and computational measures

|  |  | GCOS-CP Autonomy | GCOS-CP Control | GCOS-CP Impersonal | PCASS AOS | PCASS PC | PHQ-9 | BRS | ω2 Choice | ω2 noChoice | ω3 Choice |
| --- | --- | --- | --- | --- | --- | --- | --- | --- | --- | --- | --- |
| GCOS-CP  Control | Corr | -0.015 |  |  |  |  |  |  |  |  |  |
|  | Sig. | 0.947 |  |  |  |  |  |  |  |  |  |
| GCOS-CP  Impersonal | Corr | **-0.500** | -0.085 |  |  |  |  |  |  |  |  |
|  | Sig. | 0.021 | 0.714 |  |  |  |  |  |  |  |  |
| PCASS  AOS | Corr | 0.269 | -0.347 | -0.255 |  |  |  |  |  |  |  |
|  | Sig. | 0.238 | 0.123 | 0.264 |  |  |  |  |  |  |  |
| PCASS  PC | Corr | **0.489** | -0.003 | **-0.653** | **0.546** |  |  |  |  |  |  |
|  | Sig. | 0.024 | 0.990 | 0.001 | 0.010 |  |  |  |  |  |  |
| PHQ-9 | Corr | **-0.510** | 0.195 | **0.449** | **-0.526** | **-0.630** |  |  |  |  |  |
|  | Sig. | 0.018 | 0.397 | 0.041 | 0.014 | 0.002 |  |  |  |  |  |
| BRS | Corr | -0.006 | -0.045 | -0.235 | -0.092 | 0.237 | 0.116 |  |  |  |  |
|  | Sig. | 0.981 | 0.848 | 0.306 | 0.691 | 0.301 | 0.616 |  |  |  |  |
| ω2  Choice | Corr | -0.243 | 0.182 | 0.028 | -0.044 | 0.142 | -0.076 | 0.094 |  |  |  |
|  | Sig. | 0.289 | 0.431 | 0.905 | 0.848 | 0.539 | 0.744 | 0.686 |  |  |  |
| ω2  noChoice | Corr | -0.303 | 0.200 | 0.292 | -0.121 | -0.228 | 0.287 | -0.018 | 0.117 |  |  |
|  | Sig. | 0.182 | 0.384 | 0.199 | 0.601 | 0.319 | 0.207 | 0.938 | 0.614 |  |  |
| ω3  Choice | Corr | 0.260 | -0.113 | -0.148 | 0.035 | -0.270 | 0.092 | -0.120 | **-0.697** | -0.285 |  |
|  | Sig. | 0.256 | 0.626 | 0.523 | 0.881 | 0.237 | 0.692 | 0.606 | 0.000 | 0.210 |  |
| ω3  noChoice | Corr | 0.268 | 0.045 | -0.148 | -0.259 | 0.002 | -0.202 | -0.088 | -0.036 | **-0.758** | 0.199 |
|  | Sig. | 0.241 | 0.846 | 0.521 | 0.257 | 0.993 | 0.380 | 0.705 | 0.879 | 0.000 | 0.387 |

Table S1: Pearson correlations between clinical, psychological and computational measures. Significances are two-tailed.

## Account of the Rescorla-Wagner model for fMRI activation

As a means of validation, timecourse estimates of the learned value $\hat{V}$ assigned to each trial type (Choice and noChoice) were estimated for each participant, and used to parametrically modulate the Cue phase of each trial. Here for each Choice trial t,

$$\hat{V}_{Choice}\left( t \right)=\hat{V}_{Choice}\left( t-1 \right)+ \alpha_{Choice}\delta(t)$$

Where $\alpha_{Choice}$ is the learning rate for Choice trials, and

$$\delta\left( t \right)=R\left( t \right)-\hat{V}_{Choice}(t-1)$$

A similar parallel system is used for noChoice trials.

At the second level, the contrast of $\hat{V}_{Choice}>\hat{V}_{noChoice}$ revealed whole-brain significant activation in bilateral supplementary motor cortex, and some non-significant signal in ventral striatum:

| **Region** | **Peak MNI** | **Peak Z** | **k_E_** | **Cluster P_FWE_** |
| --- | --- | --- | --- | --- |
| R Supplementary motor cortex | 6 11 50 | 4.06 | 57 | 0.003 |
| L Supplementary motor cortex | -6 17 50 | 3.49 |  |  |
| L Putamen | -15 5 -4 | 3.96 | 25 | 0.132 |

Table S2: Regions showing whole-brain activation for the Rescorla-Wagner model-derived estimates of $\hat{V}_{Choice}>\hat{V}_{noChoice}$.


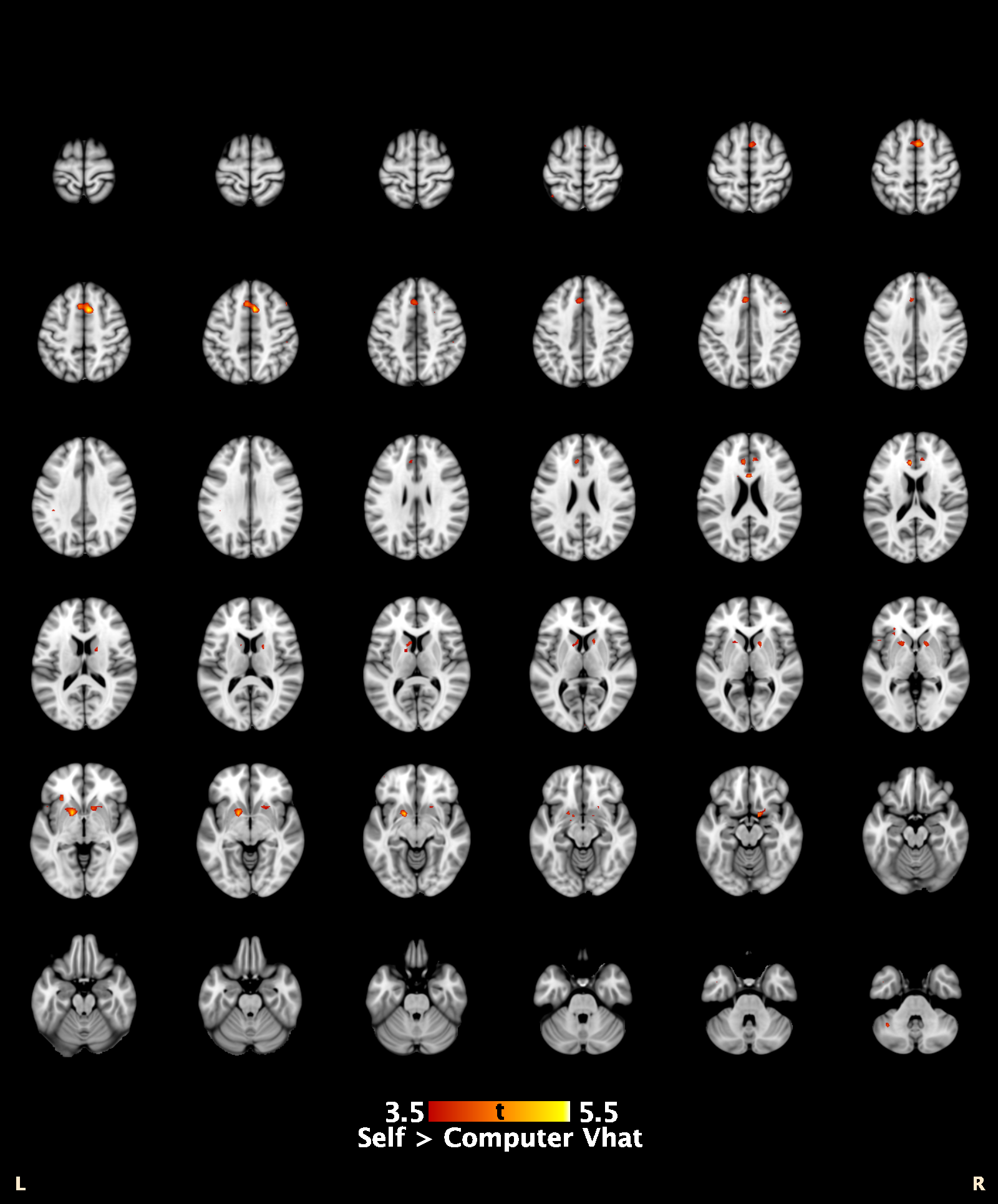


Figure S1: Activation for the Rescorla-Wagner model-derived contrast of $\hat{V}_{Choice}>\hat{V}_{noChoice}$ , displayed at an uncorrected voxelwise p threshold of < 0.001.

Although there was some overlap with the regions accounted for by the 3-layer HGF with uncertainty-dependent response model, the Rescorla-Wagner model was not able to provide as good an account for brain activation overall.
